# Gene Deregulation and Underlying Mechanisms in Spinocerebellar Ataxias With Polyglutamine Expansion

**DOI:** 10.3389/fnins.2020.00571

**Published:** 2020-06-09

**Authors:** Anna Niewiadomska-Cimicka, Antoine Hache, Yvon Trottier

**Affiliations:** ^1^Institut de Génétique et de Biologie Moléculaire et Cellulaire (IGBMC), Illkirch, France; ^2^Centre National de la Recherche Scientifique, UMR7104, Illkirch, France; ^3^Institut National de la Santé et de la Recherche Médicale, U964, Illkirch, France; ^4^Université de Strasbourg, Strasbourg, France

**Keywords:** polyglutamine, spinocerebellar ataxia, SCA, epigenetic, transcriptional dysregulation, Purkinje cells

## Abstract

Polyglutamine spinocerebellar ataxias (polyQ SCAs) include SCA1, SCA2, SCA3, SCA6, SCA7, and SCA17 and constitute a group of adult onset neurodegenerative disorders caused by the expansion of a CAG repeat sequence located within the coding region of specific genes, which translates into polyglutamine tract in the corresponding proteins. PolyQ SCAs are characterized by degeneration of the cerebellum and its associated structures and lead to progressive ataxia and other diverse symptoms. In recent years, gene and epigenetic deregulations have been shown to play a critical role in the pathogenesis of polyQ SCAs. Here, we provide an overview of the functions of wild type and pathogenic polyQ SCA proteins in gene regulation, describe the extent and nature of gene expression changes and their pathological consequences in diseases, and discuss potential avenues to further investigate converging and distinct disease pathways and to develop therapeutic strategies.

## Introduction

The dominantly inherited spinocerebellar ataxias (SCAs) represent a large and heterogeneous group of neurodegenerative diseases mainly characterized by progressive loss of balance and coordination often associated with slurred speech (Klockgether, 2011). SCA symptoms mainly arise from the progressive degeneration of the cerebellum, brainstem and associated structures. In the cerebellum, Purkinje cells are the neuronal population the most frequently affected. Other central and peripheral nervous structures can be affected and contribute to additional and diverse symptoms. The estimated prevalence of SCAs is 1–5 per 100,000 individuals, but varies markedly depending on geography and ethnicity (Ruano et al., 2014).

Among this group, SCA1, SCA2, SCA3, SCA6, SCA7, and SCA17 share a common causative mutation that is the expansion of a CAG repeat sequence located within the coding region of specific genes, which translates into a polyglutamine (polyQ) tract in the corresponding proteins: Ataxin-1 (ATXN1), ATXN2, ATXN3, the α1A subunit of the Cav2.1 voltage-gated calcium channel (CACNA1A), ATXN7, and the TATA-box-binding protein (TBP), respectively. Other polyQ disorders include Huntington’s disease (HD), the spinobulbar muscular atrophy and the dentatorubral-pallidoluysian atrophy. The normal polyQ tract in SCA proteins is generally polymorphic in length, while beyond an apparent threshold the diseases become fully penetrant. Depending on the SCA, the pathogenic properties of the mutated protein can appear with an expansion ranging from 20 to several hundreds of glutamines (Buijsen et al., 2019). PolyQ SCAs are generally adult onset disorders and progress over 10–20 years before leading to death of patients (Klockgether, 2011). However, longer polyQ expansions are associated with earlier onset of symptoms and a broader range of neurological symptoms in juvenile forms.

Despite their similarities, the polyQ SCAs differ in many aspects. While SCA proteins bear a similar expandable polyQ tract, they do not share any other domain and have different cellular functions. Even if they are ubiquitously expressed, they cause relatively distinct pattern of neurodegeneration accounting for SCA specific clinical features. Therefore, the particularities of each polyQ SCA is believed to come from the protein context into which the polyQ expansion is embedded.

The exact pathogenic mechanisms underlying polyQ SCAs are not fully understood (Buijsen et al., 2019). Current studies indicate a complex mechanism involving the disruption of the native protein functions -through gain or loss of function- and the toxic gain of function conveyed by the polyQ expansion. A major and common impact of polyQ expansion on proteins is a conformational change leading to misfolding, progressive intracellular accumulation and the formation of large insoluble protein aggregates containing the mutant protein. While it is still under debate whether large polyQ aggregates play a role in SCA pathogenesis, the aggregation is known to proceed stepwise generating small soluble oligomeric species, which are thought to induce proteotoxicity (Buijsen et al., 2019).

PolyQ SCAs pathogenesis leads to progressive alterations of several cellular functions including autophagy, ubiquitin-proteasome degradation, calcium homeostasis, neurotransmission, and mitochondrial energy production, which contribute to neuronal dyshomeostasis and progressive death (Klockgether, 2011; Meera et al., 2016; Buijsen et al., 2019). Early studies also reported that changes in gene expression are central features in polyQ SCAs. Many groups have emphasized the interactions of polyQ SCA proteins with different factors and co-regulators of gene transcription, DNA sequences and proteins involved in RNA metabolism (Table 1). Moreover, misfolded SCA proteins progressively accumulate in neuronal nuclei where they form aggregates that sequester nuclear proteins or perturb the nuclear proteostasis network. It is noteworthy that preventing the nuclear localization of polyQ SCAs often mitigates disease features in experimental model systems. Finally, changes in gene expression are observed at disease onset in different animal models of polyQ SCAs, accounting for diverse cellular dysfunctions, but also likely reflect some compensatory responses to proteotoxicity (Table 2).

**TABLE 1:** Cellular function and main interactors of polyQ SCA proteins.

**Disease**	**Causative gene/protein**	**Cellular function of disease associated protein**	**Main interactors**	**Cellular function**	**References**
SCA1	*ATXN1*/ataxin-1 (ATXN1)	Transcription; RNA metabolism	ATXN1L (BOAT)	Transcription; corepressor	Mizutani et al., 2005; Bowman et al., 2007
			CIC	Transcriptional repressor	Lim et al., 2008; Rousseaux et al., 2018
			HDAC3	Histone deacetylation, epigenetic repressor	Venkatraman et al., 2014
			LANP	Inhibitor of histone acetyltransferases	Matilla et al., 1997; Cvetanovic et al., 2007, 2012
			PQBP-1	Transcription; splicing	Okazawa et al., 2002
			RBM17	Splicing	Lim et al., 2008
			RORa/TIP60	Transcriptional activation	Serra et al., 2006
			SMRT/NCOR2	Transcriptional corepressor	Tsai et al., 2004
SCA2	*ATXN2*/ataxin-2 (ATXN2)	RNA metabolism; translation	PABP-1	mRNA splicing and stability	Satterfield and Pallanck, 2006
			A2BP1/Fox1	Splicing	Shibata et al., 2000
			DDX6	RNA degradation	Nonhoff et al., 2007
			TDP-43	RNA processing; transcription	Elden et al., 2010
SCA3	*ATXN3*/ataxin-3 (ATXN3)	Deubiquitinase; transcriptional regulator	CBP/PCAF/P300	Histone acetylation	Chai et al., 2001; Li et al., 2002
			HDAC3/NCOR	Histone deacetylation	Evert et al., 2006; Feng et al., 2018
			FOXO4	Transcriptional activation	Araujo et al., 2011
			TBP	Basal transcription	Araujo et al., 2011
			PML	Nuclear bodies	Chai et al., 2001
SCA6	*CACNA1A*/α1A subunit of Cav2.1 and α1ACT	Calcium channel subunit and transcriptional activator	AT-rich and CA-rich DNA elements	Enhancers	Du et al., 2013
SCA7	*ATXN7*/ataxin-7 (ATXN7)	SAGA component; co-activator of RNAPII-driven transcription	GCN5	Histone acetylation	Helmlinger et al., 2004
			USP22	Histone deubiquitinase	Helmlinger et al., 2004
			CRX	Transcriptional activation	La Spada et al., 2001; Chen S. et al., 2004
			RORa	Transcriptional activation	Strom et al., 2005
			SIRT1	Deacetylase	Stoyas et al., 2019
			TBP	Basal transcription	Papai et al., 2020
SCA17	*TBP*/TATA-box binding protein (TBP)	TFIID component; basal transcription	DNA elements	Promoter regions	Friedman et al., 2008
			TFIIB	General transcription	Friedman et al., 2007
			XBP1	Transcriptional activation	Yang et al., 2014
			MYOD	Transcriptional activation	Heller and Bengal, 1998
			SP1	Transcriptional activation	Shah et al., 2009

**TABLE 2:** Gene expression impairments in polyQ SCA mouse models.

**Disease**	**Mouse model name**	**Design**	**Mutant protein expression**	**Phenotypes and neuropathology**	**Deregulation of genes, pathways and epigenetic**	**References**
SCA1	SCA1[82Q]	Tg; *Pcp-2* promoter, *ATXN1* cDNA with 82 CAGs	PC; overexpress	Motor phenotype; cerebellar and PC pathology	LTD; Glutamate signaling in PC; Cell signal transduction; calcium homeostasis; multicellular impairments: PCP; IO: immune system, ISG	Lin et al., 2000; Serra et al., 2004; Ingram et al., 2016; Driessen et al., 2018
	Atxn1^154Q/2Q^	KI;154 CAGs in *Atxn1*	Ubiquitous; endogenous level	Motor phenotype; cerebellar and PC pathology	PC: cellular signaling pathways; *Igfbp5* and IGF pathway crosstalk PC-GC; medulla: metabolic pathways; IO: immune system; ISG; histone H3 hypoacetylation	Gatchel et al., 2008; Cvetanovic et al., 2012; Driessen et al., 2018
SCA2	SCA2-127Q	Tg; *Pcp-2* promoter, *ATXN2* cDNA with 127 CAGs	PC; overexpress	Motor phenotype; cerebellar and PC pathology	LTD; phosphatidylinositol; calcium homeostasis; small GTPase-mediated signaling; mRNA transport; synaptogenesis; axon guidance	Hansen et al., 2013; Pflieger et al., 2017
	BAC-Q72	Tg; BAC containing human *ATXN2* with 72 CAGs	Brain; endogenous level; overexpress in PC and basal ganglia	Motor phenotype; cerebellar and PC pathology	Calcium homeostasis; Glutamate signaling; voltage-gated ion channel activity	Dansithong et al., 2015
	Atxn2-CAG100	KI; 100 CAGs in *Atxn2*	Ubiquitous; endogenous level	Motor phenotype	Myelination	Sen et al., 2019a
SCA3	Ataxin-3-Q79	Tg; Prion promoter, *ATXN3* cDNA with 79 CAGs;	Whole brain except PC; overexpress	Motor phenotype; cerebellar and PC pathology	LTD; Glutamate signaling in PC; Cell signal transduction; calcium homeostasis; GABA receptors; gene expression regulators; histone H3 and H4 hypoacetylation	Chou et al., 2008
	YAC84Q (MJD84.2)	Tg; YAC containing *ATXN3* with 76–77 CAGs	Ubiquitous; endogenous level	No motor phenotype	CREB signaling; cholesterol biosynthesis; myelination; striatum: axon guidance; neurotransmission brainstem: cell signaling; aa metabolism	Toonen et al., 2018
	SCA3	KI; 82 CAGs in *Atxn3*	Ubiquitous; endogenous level	No motor phenotype	Myelination	Ramani et al., 2017
SCA6	PCαACT_SCA6_	Tg; *Pcp-2* promoter, *α1ACT* cDNA with 33 CAGs	PC; overexpress	Weak motor phenotype; cerebellar and PC pathology	Neurite outgrowth; neuronal differentiation	Du et al., 2013
	SCA6-MPI^118Q/118Q^	KI; 118 CAGs in *Cacna1a*	Ubiquitous; endogenous level	Motor phenotype; cerebellar and PC pathology	GPCR signaling; Neuroinflammation	Aikawa et al., 2015
SCA7	R7E	Tg; *Rho promoter*, *ATXN7* cDNA with 90 CAGs	Rod photoreceptors; overexpress	Photoreceptor dysfunction and dystrophy	Phototransduction pathway; H3 hypoacetylation	Abou-Sleymane et al., 2006
	ataxin-7-Q52	Tg; PDGF-B promoter, *ATXN7* cDNA with 52 CAGs	Brain; overexpress	Motor phenotype; cerebellar and PC pathology	Myelination; synaptic transmission; signal transduction; axon transport; neuronal differentiation	Chou et al., 2010
	PrP-SCA7-92Q	Tg; Prion promoter, *ATXN7* cDNA with 92 CAGs	Whole brain except PC; overexpress	Photoreceptor dysfunction and dystrophy; Motor phenotype; cerebellar and PC pathology	Phototransduction pathway; H3 hypoacetylation	La Spada et al., 2001; Palhan et al., 2005
	Gfa2-SCA7-92Q	Tg; *Gfa2* promoter, *ATXN7* cDNA with 92 CAGs	Bergmann glia	Motor phenotype; cerebellar and PC pathology	Glutamate transporter genes	Custer et al., 2006
	mPrP-fx SCA7-92Q BAC	Tg; Prion promoter in BAC *ATXN7* cDNA with 92 CAGs; eGFP	Whole brain; endogenous level	Motor phenotype; cerebellum and PC pathology	Phosphatidylinositol and calcium signaling	Stoyas et al., 2019
	SCA7^266Q/5Q^	KI; 266 CAGs in *Atxn7*	Ubiquitous; endogenous level	Photoreceptor dysfunction and dystrophy; motor phenotype; cerebellar and PC pathology	Phototransduction pathway; transcription factor; MHC class I; cell signaling; *Igfbp5* and IGF pathway	Yoo et al., 2003; Gatchel et al., 2008
SCA17	TBP-71Q/TBP-105Q	Tg; Prion promoter, *TBP* cDNA with 71 or 105 CAGs	Whole brain except PC; overexpress	Motor phenotype; cerebellar and PC pathology	Downregulation of *Hspb1* and *HSPA5*	Friedman et al., 2007; Huang et al., 2011
	TBP-105Q	KI; 105 CAGs in *Tbp*	Ubiquitous; endogenous level	Motor phenotype; cerebellar and PC pathology skeletal muscle phenotype	Synaptic transmission; calcium signaling; immune system; muscle specific genes	Yang et al., 2014; Huang S. et al., 2015; Liu et al., 2020

**Tg, transgenic; KI, knockin; YAC, yeast artificial chromosome; BAC, bacterial artificial chromosome; PC, Purkinje cells; GC, granule cells; IO, inferior olive; GPCR, G-protein coupled receptor; PCP, planar cell polarity; ISG, interferon stimulated gene; aa, amino acid.**

In this review, we summarize our current understanding of gene deregulation mechanisms and consequences in the pathogenesis of polyQ SCAs. We also highlight the still poorly investigated roles of epigenetic mechanisms and cell-type specific regulatory networks and we bring up some critical questions such as the role of transcription buffering, nuclear protein quality controls (nPQC) and aging to elucidate the disease-specific and common pathomechanisms of polyQ SCAs.

## Deregulation of Gene Expression and Epigenetic

### SCA1

SCA1 is caused by the expansion of CAG triplets to 39 or more repeats in the *ATXN1* gene, which translates into an expanded polyQ domain in ATXN1 (Orr et al., 1993). The disease progresses the most rapidly within polyQ SCA subtypes (Jacobi et al., 2015), causing a progressive gait ataxia, dysarthria, neuropathies, nystagmus and slurred speech (Zoghbi, 1995; Klockgether, 2011). Although wild type ATXN1 is widely expressed, mutant ATXN1 (mATXN1) causes olivopontocerebellar atrophy with selective degeneration of Purkinje cells and neurons in the deep cerebellar nuclei, the brainstem and the spinal cord (Zoghbi, 1995; Klockgether, 2011).

ATXN1 localizes to the nucleus and is mainly known for its repressor function in gene expression (Klement et al., 1998; Tsai et al., 2004; Lam et al., 2006; Bolger et al., 2007; Venkatraman et al., 2014). ATXN1 interacts with multiple nuclear proteins involved in transcription regulation such as HDAC3, RORa/TIP60 complex, CIC, LANP, SMRT/NCOR2 complex, HDAC4/MEF2 complex, ATXN1L (BOAT), GFI-1, 14-3-3, Sp1, A1Up, PQBP1 (Matilla et al., 1997; Davidson et al., 2000; Okazawa et al., 2002; Chen et al., 2003; Tsai et al., 2004; Mizutani et al., 2005; Tsuda et al., 2005; Lam et al., 2006; Serra et al., 2006; Bolger et al., 2007; Goold et al., 2007; Lim et al., 2008), and also with the RNA-binding protein RBM17 (Yue et al., 2001; Chen Y. W. et al., 2004; Lim et al., 2008). Some of these interactions are mediated by the HMG-box protein 1 (AXH) domain of ATXN1 and are modulated by the phosphorylation of its serine 776 (Ser776) (Yue et al., 2001; Chen Y. W. et al., 2004; Tsai et al., 2004; Mizutani et al., 2005; Tsuda et al., 2005; Lam et al., 2006; Serra et al., 2006; Lim et al., 2008; Gehrking et al., 2011). mATXN1 shows altered interactions with many of these natural partners, leading to gene deregulation and neuronal degeneration in SCA1 cellular and mouse models. In addition, mATXN1 accumulates and aggregates in neuronal nuclei of SCA1 patients’ brain and model systems (Skinner et al., 1997; Cummings et al., 1998), and sequesters interactors, which accounts for their dysfunction. Importantly, gene deregulations precede behavioral impairments in mouse models (Lin et al., 2000; Serra et al., 2004) and mutation in mATXN1 nuclear localization signal abolishes the phenotypes, pointing out the nuclear origin of the toxicity (Klement et al., 1998).

Studies on gene deregulation in SCA1 are rich and cover multiple aspects. One of the first mechanism proposed to alter gene expression was through the dysfunction of PQBP1, a binding partner of RNA polymerase II (RNAPII) enriched in brain regions degenerating in SCA1. The expression of mATXN1 in cultured cells increases the binding of PQBP1 to RNAPII, diminishing the level of phosphorylated form of RNAPII, and hence reducing transcription (Okazawa et al., 2002). Consistently, the phosphorylation of RNAPII is decreased in the cerebellar cortex of SCA1[82Q] transgenic mice (Okazawa et al., 2002), suggesting that transcription elongation mediated by phospho-RNAPII may be impaired in the disease.

Several evidences indicate that mATXN1 affects the genetic program of Purkinje cells development and maintenance. Firstly, SCA1[82Q] transgenic mice expressing mATXN1 specifically in Purkinje cells show that behavioral phenotypes occur after the Purkinje cells pathology, which is characterized by the downregulation of Purkinje cells genes involved in glutamate signaling, long term depression (LTD) and calcium homeostasis (e.g., *Itpr1*, *Serca2*, *Eaat4*, *Trpc3*, and *Inpp5A*) (Lin et al., 2000; Serra et al., 2004). SCA1[82Q] transgenic mice in which mATXN1 conditional expression was induced after completion of cerebellar development, present milder phenotypes than mice expressing mATXN1 during post-natal cerebellar development (Zu et al., 2004; Serra et al., 2006). Secondly, the expression level of retinoid acid receptor-related orphan receptor alpha (RORa) is decreased in the cerebellum of SCA1[82Q] transgenic mice. RORa is crucial for the development and maintenance of Purkinje cells and *Rora* gene mutation in *Staggerer* mice leads to cerebellar defects and ataxia (Hamilton et al., 1996; Serra et al., 2006). SCA1[82Q] transgenic and *Staggerer* mice share common deregulated genes involved in calcium and glutamate signaling (Lin et al., 2000; Serra et al., 2004). A loss-of-function mutation for several of these genes such as *Calb1, Itpr1, Grm1* causes ataxia (Hoxha et al., 2018). Consistently, RORa haploinsufficiency in SCA1[82Q] transgenic mice worsens the pathology (Serra et al., 2006). Thirdly, ATXN1 interacts with the Tat Interacting Protein, 60 kDa (TIP60, also named KAT5), an histone acetyltransferase (HAT), to form a complex with RORa. PolyQ expansion in ATXN1 reinforces the interactions with TIP60 (Serra et al., 2006). Partial loss of TIP60 delays cerebellar degeneration in SCA1[82Q] transgenic mice and correlates with an increased level of RORa and RORa-dependent gene expression (Gehrking et al., 2011). Therefore, these results suggest a mechanism whereby increased interactions of mATXN1 with TIP60 destabilizes RORa transactivation activity, leading to reduced expression of genes important for the function and maintenance of Purkinje cells.

To further delineate the role of gene deregulation in Purkinje cells pathology, SCA1[82Q] mice were studied at mild, moderate and severe ataxia stages (Ingram et al., 2016), and showed that downregulated genes are overrepresented at the mild stage, while the severe stage is associated with gene upregulation. Weighted gene co-expression network analysis (WGCNA) of cerebellar transcriptomes identified 2 modules of deregulated genes highly relevant for SCA1 ataxia phenotype: the so called Magenta module (MM) reflects an early and progressive signature of downregulated Purkinje cells genes, while Lt Yellow module (LtYM) concerns up- and downregulated genes associated with multicellular responses to Purkinje cells pathology (Ingram et al., 2016). Ingenuity Pathway Analysis (IPA) of MM identified ATXN1 as the main upstream regulator of MM genes and highlighted the function of these genes in LTD and glutamate receptor signaling. The most connected hub genes in MM module include *Fam107b, Fam21*, *Gabbr1*, *Rgs8, Dner, Inpp5a, Trpc3, Homer3, Stac, and Grik1* and many of them code for signal transduction proteins. Importantly, SCA1^154Q/2Q^ knockin mice also show similar downregulation of Purkinje cell genes including several hub genes. Finally, promoter analysis of MM genes shows an enrichment for motifs bound by the Capicua Transcriptional Repressor (CIC), which interacts with ATXN1 (see below) (Ingram et al., 2016). Together, this analysis identified a set of deregulated genes and underlying mechanisms that compromise the functions of Purkinje cells in SCA1.

A series of studies conducted in mouse models have highlighted the complex interplay between gain- and loss-of-function mechanisms at the basis of gene deregulation in SCA1. *Atxn1* knockout mice exhibit a variety of neurological deficits including motor learning, a phenotype shared with SCA1^154Q/2Q^ knockin mice (Matilla et al., 1998; Crespo-Barreto et al., 2010). Furthermore, comparison of cerebellar transcriptomes reveals common genes deregulated in the two models (Crespo-Barreto et al., 2010), suggesting that loss of ATXN1 function might contribute in part to SCA1 pathogenesis. Along this line, removing the wild-type copy of ATXN1 in SCA1^154Q/2Q^ mice worsens the disease phenotype (Lim et al., 2008), while duplication of *Ataxin-1-Like* gene (*Atxn1l*), coding for a highly conserved *Atxn1* paralog with partial functional redundancy (Crespo-Barreto et al., 2010), suppresses behavioral phenotypes in SCA1^154Q/2Q^ mice (Bowman et al., 2007), raising the possibility that ATXN1L replaces mATXN1 in native complexes. In particular, ATXN1 forms a complex with CIC in mouse cerebellum, while mATXN1 decreases the formation of the native complex (Lim et al., 2008) and alters the repressor activity of CIC *in vitro* and *in vivo* (Lam et al., 2006). Therefore, it is proposed that increased ATXN1L level by *Atxn1l* duplication in SCA1 mice displaces mATXN1 from the CIC complex, promotes its aggregation and thus restores functional ATXN1L-CIC repressor activity (Bowman et al., 2007). The role of CIC in SCA1 was further explored by Fryer et al. (2011), who showed that increased physical activity does not improve motor phenotypes in SCA1^154Q/2Q^ knockin mice, but prolongs their survival in correlation with a decreased CIC level in brainstem (Fryer et al., 2011). Furthermore, they found that deletion of one allele of CIC (CIC-L^+/–^) in SCA1^154Q/2Q^ knockin mice leads to multiple beneficial effects on motor coordination, learning and memory, Purkinje cells loss, weight loss and survival (Fryer et al., 2011). Analyses of cerebellar transcriptomes and direct CIC targets identified by chromatin immunoprecipitation (ChIP) led to the conclusion that mATXN1 causes a dual effect on DNA binding properties of CIC, which binds either more tightly and hyper-repressed certain gene targets or more weakly to other targets and leads to transcriptional derepression. Together, these results indicate that mATXN1 causes concomitant gain- and loss-of-function of the same native partner. A more recent study indicates that gain-of-function of mATXN1-CIC complex might be the major factor inducing cerebellar pathology (Rousseaux et al., 2018). Indeed, complete deletion of CIC in the cerebellum does not cause much gene deregulation, or Purkinje cells degeneration. Moreover, the expression of mATXN1 with a mutation that prevents interaction with CIC ameliorates SCA1 symptoms and reduces gene deregulations in the cerebellum of SCA1[82Q] mice (Rousseaux et al., 2018). In the mouse cerebellum, ATXN1 is present in another native complex containing RBM17 and is thought to be involved in RNA splicing (Lim et al., 2008). PolyQ expansion leads to increased association with the RMB17 complex. Interestingly, loss of one *dRbm17* allele suppresses mATXN1 toxicity in the SCA1 *Drosophila* eye and overexpression of the human RBM17 worsens the phenotype, consistent with another gain-of-function mechanism. This further suggests the involvement of RNA splicing dysfunction in the pathogenesis.

Gene deregulation in SCA1 may also result from epigenetic alterations, as SCA1^154Q/2Q^ knockin mouse cerebella show a significant reduction of histone H3 acetylation, an epigenetic modification associated with gene activation (Cvetanovic et al., 2012). ATXN1 interacts with a transcriptional repressor complex formed by SMRT, NCOR2, and the histone deacetylase HDAC3 (Tsai et al., 2004; Emmett and Lazar, 2019), while mATXN1 stabilizes this complex and favors transcriptional repression (Venkatraman et al., 2014). However, deleting a single allele of HDAC3 in SCA1^154Q/2Q^ knockin mice has neither beneficial effects on motor deficits nor on Purkinje cell pathology, likely because HDAC3 is required for Purkinje cell functions as shown in conditional HDAC3 knockout mice (Venkatraman et al., 2014). This confounding effect does not rule out HDAC3 dysfunction, but precludes pharmacological inhibition of HDAC3 in therapy of SCA1 and other polyQ SCAs (Venkatraman et al., 2014). mATXN1 also affects the function of LANP, a protein enriched in Purkinje cells that binds the N-terminal histone tails and prevents its acetylation by HATs (Seo et al., 2002; Cvetanovic et al., 2007). mATXN1 perturbs a repressor complex formed by LANP and E4F, and relieves the transcriptional repression dependent of this complex (Matilla et al., 1997; Cvetanovic et al., 2007). In addition, mATXN1 interacts and synergises with LANP to inhibit the HAT activity of CBP and to decrease transcription in N2A cells (Cvetanovic et al., 2012). Consistently, LANP depletion in these cells restores the histone acetylation level. Inactivation of *Lanp* in SCA1^154Q/2Q^ knockin mice improves the maintenance of synapses between the inferior olive (IO) neurons and Purkinje cells as measured by vGLUT2 labeling. However, this improvement is not sufficient to restore behavioral deficits, suggesting that LANP might have cell-type specific functions.

Recent studies have explored gene deregulation in extra cerebellar regions. In the medulla of SCA1^154Q/2Q^ knockin mice, deregulation occurs 10 weeks later than in the cerebellum, and concerns genes mainly involved in metabolic pathways (Friedrich et al., 2018). Interestingly, gene deregulation in SCA1^154Q/2Q^ mouse IO nuclei, which are highly affected in SCA1 *post-mortem* tissues, reveals the activation of the immune system including interferon stimulated genes (ISG) (Driessen et al., 2018). Similar immune response activation is observed in SCA1[82Q] transgenic mice, despite the absence of mATXN1 expression in IO, suggesting an underlying non-cell autonomous mechanism (Driessen et al., 2018). The identification of *Igfbp5* as the most robustly downregulated gene in SCA1^154Q/2Q^ and SCA7^266Q/5Q^ knockin mice revealed another non-cell autonomous mechanism (Watase et al., 2002; Yoo et al., 2003). *Igfbp5* is expressed and secreted from granule neurons, and is thought to play a role in the regulation of the insulin-like growth factor (IGF) pathway by blocking the binding of IGF1 to its receptor. In SCA1 and SCA7 mouse models, the decreased level of IGFBP5 leads to the activation of IGF pathway (Gatchel et al., 2008). Interestingly, *Igfpb5* is also downregulated in granule neurons of SCA1[82Q] transgenic mice, despite the absence of mATXN1 expression in these neurons (Burright et al., 1995), leading to the abnormal activation of IGF pathway in the Purkinje cells of this model. Overall, this indicates that Purkinje cells pathology in SCA1[82Q] mice causes the downregulation of *Igfpb5* in granule neurons in a non-cell autonomous manner, and in turn the decreased IGFBP5 level produced by granule neurons leads to the abnormal activation of the IGF pathway in Purkinje cells. As a consequence, IGF downstream signaling leads to the activation of PI3-K/Akt (Gatchel et al., 2008), which can phosphorylate the Ser776 of mATXN1 and enhance its toxicity (Chen et al., 2003).

### SCA2

SCA2 is caused by the expansion of a CAG triplet ranging from 33 to 500 repeats in the coding sequence of the *ATXN2* gene (Imbert et al., 1996; Pulst et al., 1996; Sanpei et al., 1996) leading to a polyQ expansion in mutant ATXN2 (mATXN2) (Pulst et al., 1996). Patients suffer from ataxia, parkinsonism, dysarthria, dysmetria, action tremor, hypotonia, and slow saccades (Buijsen et al., 2019). Several structures like brainstem, spinal cord, thalamus, cranial nerves, and muscles are affected (Rub et al., 2003; Lastres-Becker et al., 2008b), but the progressive atrophy of the cerebellum with Purkinje cell death is the primarily sign of neurodegeneration (Estrada et al., 1999; Hansen et al., 2013; Alves-Cruzeiro et al., 2016).

ATXN2 is a cytoplasmic protein widely expressed in the brain (Pulst et al., 1996). mATXN2 accumulates in the cytoplasm and nucleus and forms aggregates in SCA2 (Seidel et al., 2017). ATXN2 is implicated in RNA metabolism including stability, translation, and degradation (Nonhoff et al., 2007; Egorova and Bezprozvanny, 2019). It contains an RNA binding Lsm domain functionally relevant to pre-mRNA splicing and mRNA decay (Ralser et al., 2005). ATXN2 assembles with ribosomes on the endoplasmic reticulum (ER) and interacts with the cytoplasmic poly(A)-binding protein 1 (PABP-1), a factor involved in translation initiation (Satterfield and Pallanck, 2006; van de Loo et al., 2009). It also interacts with the RNA splicing factor A2BP1/Fox1, DDX6, and TDP-43 (Shibata et al., 2000; Nonhoff et al., 2007; Elden et al., 2010). ATXN2 localizes to stress granules arresting untranslated mRNAs under cellular stress (Nonhoff et al., 2007).

Studies in mouse models have shown the role of gene deregulation in the pathogenesis of SCA2. It is noteworthy that *Atxn2*^–/–^ KO mice develop obesity and insulin resistance and show gene deregulation in the cerebellum, despite the absence of cerebellar pathology (Kiehl et al., 2006; Lastres-Becker et al., 2008a; Hansen et al., 2013; Halbach et al., 2017; Pflieger et al., 2017). The transgenic SCA2^127Q^ mice, which express mATXN2 specifically in Purkinje cells, exhibit deregulation of genes involved in calcium signaling and changes in the firing frequency of Purkinje cells, prior to the development of a severe ataxic phenotype (Hansen et al., 2013). Further temporal analysis shows that deregulation of genes involved in histone acetylation, chromatin remodeling and cell adhesion occurs as early as post-natal day 1, while genes involved in calcium signaling and glutamatergic input important for Purkinje cells are deregulated later (Pflieger et al., 2017). Interestingly, as for SCA1 mice, WGCNA also identified a module enriched in Purkinje cells genes involved in LTD, phosphatidylinositol, calcium and signal transduction, including the hub genes *Fam21*, *Gabbr1*, *Rgs8*, and *Lcmt*. Similar deregulation of Purkinje cells genes occurs in BAC-72Q transgenic mice, which express m*ATXN2* gene in a bacterial artificial chromosome (BAC) and show Purkinje cells pathology that precedes motor deficits (Dansithong et al., 2015). Deregulation also concerns genes expressed in cerebellar granule cells, indicating that mATXN2 causes dysfunction of this cell type as well (Dansithong et al., 2015).

mATXN2 alters gene expression in part through its dysfunctions in RNA metabolism. In BAC-Q72 mice, the level of *Rgs8* mRNA is reduced, while the level of RGS8 proteins is even more strongly decreased (Dansithong et al., 2015). RGS8 is a regulator of G-protein that negatively modulates GPCR signaling and its deregulation is thought to account for the deficit of the mGluR1-ITPR1 axis in SCA2 Purkinje cells (Meera et al., 2016). It was shown that ATXN2 interacts with *Rgs8* mRNA to control its translation and the protein production *in vitro*, whereas mATXN2 has decreased interaction with *Rgs8* mRNA resulting in reduced RGS8 synthesis. Interestingly, an antisense oligonucleotide (ASO) treatment lowers the level of mATXN2 and decreases the Purkinje cell pathology in SCA2 mouse models (Scoles et al., 2017). The expression of 6 deregulated genes is restored to normal protein levels, while mRNA levels are restored for only 3 of them (Scoles et al., 2017), suggesting that ASO primarily rescues the dysfunction of mATXN2 in RNA translation, and has partial effect on other mechanisms altering the mRNA levels (Dansithong et al., 2015; Fittschen et al., 2015).

Consistent with the demyelination reported in the brain of SCA2 patients (Rub et al., 2013), lipid profiling analysis of SCA2 patient cerebellum shows deficits for very long-chain C24 sphingomyelin, a lipid enriched in brain white matter, as well as galactosylceramide, cholesterol and other lipidic compounds typical of myelin sheets (Sen et al., 2019a, b). Similarly, the cerebellum and spinal cord of Atxn2-CAG100 knockin mice show a reduction of the very long-chain sphingomyelin and free ceramide species, which correlates, respectively, with the downregulation of elongase genes (*Elovl1*, *Elovl4*, and *Elovl5)* and of the *Cers1* gene involved in their synthesis (Sen et al., 2019a). Therefore, the study establishes a link between the alteration of the white matter and specific gene deregulation (Sen et al., 2019a).

### SCA3

SCA3 is the most common dominantly inherited form of ataxia, affecting approximately 1:50,000–100,000 people (Durr, 2010). SCA3 neuropathology is characterized by degeneration in the brainstem, cerebellum, striatum, midbrain, thalamus, and spinal cord (Rüb et al., 2013). Compared to other ataxias, the Purkinje cells are relatively well preserved, except in the cerebellar vermis. The pathology primarily affects the basis pontis and deep cerebellar nuclei resulting in enlargement of the fourth ventricule (Rüb et al., 2013).

ATXN3 is an ubiquitously expressed, nucleo-cytoplasmic protein with deubiquitinase (DUB) activity (Paulson et al., 1997; Burnett et al., 2003) (and reviewed in Costa and Paulson, 2012). It is proposed to play a role in PQC regulation in the cytoplasm by editing polyubiquitin chains of substrates prior to proteasome degradation or by removing polyubiquitin chains to protect some substrates from degradation (Chai et al., 2004; Schmitt et al., 2007; Wang et al., 2012; Liu et al., 2016). The DUB activity has additional nuclear functions, such as DNA damage repair (reviewed in McLoughlin et al., 2020) and transcriptional regulation. The protein interacts with several transcriptional regulators including the HAT proteins CBP, P300, PCAF, and HDAC1-3 as well as PML, TBP, FOXO4, TAF130, MAML1, SC35, and NCoR (Chai et al., 2001; Li et al., 2002; Evert et al., 2006; Araujo et al., 2011). In particular, ATXN3 shows a dual effect on HDAC3 function in transcription. On the one hand, ATXN3, HDAC3, and NCoR form a deacetylation complex to repress transcription of the MMP2 gene (Evert et al., 2006). On the other hand, during viral infection, ATXN3 DUB activity protects HDAC3 from degradation to activate the interferon I pathway (Feng et al., 2018). In addition, upon heat and oxidative stress, ATXN3 activates FOXO4-mediated SOD2 expression (Araujo et al., 2011). Therefore, the role of ATXN3 in gene regulation might be related to cellular response to adverse conditions, such as viral infection and stress. Accordingly, in normal husbandry condition, *Atxn3* knockout mice show only a small subset of deregulated genes (Ramani et al., 2017) and appear phenotypically normal (Reina et al., 2010).

One major consequence of polyQ expansion in ATXN3 is the abnormal protein accumulation and aggregation in neuronal nuclei. Preventing nuclear localization of mutant ATXN3 (mATXN3) mitigates many disease features, while targeting mATXN3 into the nucleus worsens the phenotype of SCA3 mice (Bichelmeier et al., 2007). PolyQ expansion does not affect the DUB activity *in vitro* (Winborn et al., 2008). However, mATXN3 nuclear accumulation perturbs the deacetylation activity of HDAC3-NCoR complex leading to higher expression of *MMP-2* gene (Evert et al., 2006), and compromises the FOXO4-mediated SOD2 expression during oxidative stress (Araujo et al., 2011). Consistently, SOD2 is downregulated in the pons of SCA3 patients, which might increase the sensitivity of this brain region to stress.

Alterations in gene expression are reported in several cellular and mouse models of SCA3 (Chai et al., 2001; Li et al., 2002; Evert et al., 2006; Chou et al., 2008; Araujo et al., 2011; Switonski et al., 2015; Ramani et al., 2017; Toonen et al., 2018). However, the extent and nature of gene expression changes and their pathological consequences largely vary between mouse models. Transgenic mice overexpressing *ATXN3* cDNA (Ataxin-3-Q79 mice) show motor defects and the deregulation of genes involved in synaptic plasticity correlates with LTD impairment in Purkinje cells (Chou et al., 2008, 2014). In this model, Purkinje cells do not express mATXN3, suggesting that dysfunction occurs through deleterious effect from pre-synaptic neurons (Chou et al., 2008). In contrast to Ataxin-3-Q79 mice, models expressing physiological levels of mATXN3 do not present motor phenotype. However, since these models show polyQ aggregation and altered gene expression, they are considered as pre-symptomatic models of SCA3. The transgenic YAC84Q (MJD84.2) SCA3 mouse model expressing a yeast artificial chromosome (YAC) containing the *ATXN3* human gene with 76-77 CAG repeat expansion, shows gene deregulation in diverse brain tissues affected in SCA3 patients, such as the striatum, brainstem, cerebral cortex and cerebellum (Toonen et al., 2018). Interestingly, these tissues consistently show downregulation of the CREB signaling pathway (Toonen et al., 2018), in agreement with previous studies showing that mATXN3 aggregates sequester and inhibit CBP, a major transcriptional co-regulator of CREB-mediated transcription (McCampbell et al., 2000; Chai et al., 2001).

In a compelling study, Ramani et al. (2017) compared alterations of gene expression in the brainstem of different SCA3 mouse models including YAC84Q SCA3 mice, its control counterpart YAC15Q, SCA3 knockin mice with 82 CAGs, and *Atxn3* knockout mice. The mouse lines show different load of mATXN3 aggregation in the brain and the comparison provides four important information (Ramani et al., 2017). Firstly, overexpression of human wild type *ATXN3* in YAC15 has little effect on gene expression and further supports its minor role in gene regulation when mice are raised in normal conditions. Secondly, the level of deregulation increases with the aggregation load in the pons of different lines, supporting a mechanism whereby mATXN3 affects the function of transcriptional regulators through sequestration in polyQ aggregates or by alteration of nuclear proteostasis (further discussed below). Thirdly, differentially expressed genes in SCA3 knockins largely differ from those in the knockout mice, implying that deregulation in SCA3 mice is not related to loss of ATXN3 function. Finally, a subset of shared genes affected in the different SCA3 mouse lines highlights alterations in the homeostasis of oligodendrocytes, where mATXN3 also forms aggregates (Ramani et al., 2017). Furthermore, conditional deletion of *mAtxn3* in oligodendrocytes rescues gene deregulation in these cells, revealing cell-autonomous effects of mAtxn3 toxicity in this cell type.

Consistent with the impairment of HAT and HDAC functions, several SCA3 models present a decrease of the bulk level of histones H3 and H4 acetylation (Yi et al., 2013; Chou et al., 2014; Lin et al., 2014; Wang et al., 2018). Moreover, downregulated LTD-associated genes in the Ataxin-3-Q79 mice have hypoacetylated promoters, and cerebellar protein extracts show a reduction of HAT activity, but not HDAC activity *in vitro* (Chou et al., 2014). Treatment with the HDAC inhibitor (HDACi) sodium butyrate rescues H3 and H4 acetylation, expression of LTD-associated genes, LTD function of Purkinje cells and mice survival (Chou et al., 2008, 2011, 2014). Similarly, other HDACi, such as valproic acid or divalproex sodium, restore the histone acetylation level and prevent cytotoxicity in SCA3 cellular models (Yi et al., 2013; Lin et al., 2014; Wang et al., 2018) and extend the survival of a SCA3 fly model (Yi et al., 2013). At variance, valproic acid treatment of CMVMJD135 mice, which overexpress mutant *ATXN3* cDNA, only mildly attenuates the motor phenotypes (Esteves et al., 2015). However, the effects of HDACi treatments need to be interpreted with caution, since they have additional effects. For instance, valproate semisodium prevents the nuclear transport of mATXN3 in cellular models, hence reducing nuclear toxicity (Wang et al., 2018), and valproic acid-treated CMVMJD135 mice show an increased expression of the ER chaperon BIP/GRP78, which may enhance the folding capacity of the ER and provide neuroprotective effects (Esteves et al., 2015).

### SCA6

Compared to other polyQ SCAs, SCA6 is considered a pure cerebellar ataxia with later onset and slower progression (Durr, 2010). SCA6 patients have a normal lifespan (Stevanin et al., 1997). Brain analysis shows cerebellar atrophy due to Purkinje cells degeneration (Yang et al., 2000), while other regions are less affected. The ataxia is caused by short expansions ranging from 20 to 33 CAGs in the *CACNA1A* gene, resulting in polyQ expansions in the α1A subunit of P/Q type calcium channel Cav2.1 (Zhuchenko et al., 1997). Cav2.1 is highly expressed in the cerebellum, particularly in Purkinje cells. Targeted disruption of mouse *Cacna1a* leads to gross neurological phenotype and ataxia soon after birth and to death at post-natal days 18–21 (Jun et al., 1999). Mutations in human *CACNA1A* cause episodic ataxia type 2 (EA2) and familial hemiplegic migraine type 1 (FHM1), which have distinct clinical presentation from SCA6 (Guida et al., 2001; Tottene et al., 2002) and have been linked to altered calcium channel function, consistent with channelopathies. The polyQ stretch is located in the cytoplasmic C-terminus tail of the α1A subunit, which also contains residues involved in channel function and regulation (Catterall, 2000). Therefore, many studies on SCA6 have focused on the channel function in the presence of polyQ expansion, but they generated conflictual results (review in Giunti et al., 2015). In any case, the apparently normal calcium channel function observed in two knockin mouse studies weakens the channelopathy hypothesis (Saegusa et al., 2007; Watase et al., 2008). SCA6 mutation with 30Q causes mild clinical symptoms in patients, and no motor phenotype in knockin mice (Watase et al., 2008). The 30Q expansion might not be toxic enough to cause ataxia during the lifespan of mice, in contrast to the hyper-expanded 84Q, which produces polyQ aggregates and ataxia in another knockin mouse model (Watase et al., 2008).

Interestingly, cumulative evidence indicates that the C-terminal tail of α1A plays a physiological role as a free polypeptide in the pathogenesis. It was initially reported that α1A subunit is cleaved to release a stable C-terminal fragment that translocates to the nuclei of Purkinje cells (Kubodera et al., 2003; Kordasiewicz et al., 2006). The polyQ expansion does not influence the cleavage and nuclear localization, but confers cytotoxic properties to the fragment. Of greater interest, Du et al. (2013) found that the *CACNA1A* transcript is bicistronic and harbors a functional internal ribosomal entry site (IRES) and encodes two proteins, the α1A subunit and a transcription factor named α1ACT, which corresponds to the C-terminal tail. Several evidence shows that α1ACT localizes to the nucleus and activates the expression of genes involved in neurite outgrowth and Purkinje cells development such as *TAF1*, *BTG1, PMCA2*, and *GRN*. Overexpression of wild type α1ACT_wt_ partially rescues the severe phenotype of *Cacna1a* knockout mice. α1ACT harboring the SCA6 mutation (α1ACT_sca6_) also translocates to the nucleus but loses the capacity to activate the expression of these Purkinje cells genes and to rescue *Cacna1a* knockout mice (Du et al., 2013). Importantly, the overexpression of α1ACT_sca6_, but not the wild type form causes a mild ataxia and cerebellar cortical atrophy in transgenic PC-α1ACT_sca6_ mice (Du et al., 2013). Therefore, rather than a channelopathy, the pathogenesis of SCA6 appears to be related to gene deregulation and to share the pathomechanisms of other polyQ SCAs.

It is noteworthy that SCA6 patient-derived induced pluripotent stem cells (iPSCs) provides an excellent model of the pathogenesis (Ishida et al., 2016). SCA6 iPSCs differentiate into Purkinje cells with the same efficiency as control iPSCs. Similar to observations in patients (Ishikawa et al., 1999), the α1A subunit abnormally accumulates in SCA6 Purkinje cells. At the opposite, the nuclear α1ACT is found at a much lower level in SCA6 Purkinje cells compared to control, correlating with the decreased expression of *TAF1* and *BTG1* mRNA and proteins (Ishida et al., 2016). Compared to controls, the *in vitro* maintenance of SCA6 Purkinje cells depends of thyroid hormone T3 in the medium. However, the relationship between T3-dependent maintenance of Purkinje cells and the decreased expression of Purkinje cell genes such as *TAF1* and *BTG1* remains to be investigated.

### SCA7

SCA7 is due to CAG expansions ranging from 34 to more than 400 triplets in the gene coding for ATXN7 (David et al., 1997; Buijsen et al., 2019). The main symptoms of SCA7 are gait and stance ataxia which are often accompanied by dysphagia. Additional symptoms include spastic ataxia, intentional tremors, slow eye movement, ophthalmoplegia, as well as pyramidal signs. These symptoms are caused by the loss of Purkinje cells and additional neuronal loss in the granular cell layer, the dentate nuclei, the IO nuclei as well as a mild neuronal loss in the basis pontis, and an atrophy of the spinocerebellar tracts (Michalik et al., 2004; Rub et al., 2005). SCA7 is the only polyQ SCA to cause cone and rod photoreceptor dystrophy responsible for a decrease of visual acuity (Michalik et al., 2004).

ATXN7 is a ubiquitously expressed nucleo-cytoplasmic protein, which has an important nuclear function as a component of the Spt-Ada-Gcn5 Acetyltransferase complex (SAGA) (Scheel et al., 2003; Helmlinger et al., 2004; Lee et al., 2009; Papai et al., 2020). SAGA harbors 2 enzymatic modules: a HAT module containing the acetyltransferases KAT2A/GCN5 or KAT2B/PCAF, involved in the acetylation of lysine 9 of histone H3 (H3K9) (Bonnet et al., 2014; Li et al., 2017), and a DUB module containing the deubiquitinase USP22, which removes monoubiquitin from lysine 120 of histone H2B (Bonnet et al., 2014). ATXN7 is part of the DUB module, together with USP22, ENY2, and ATXN7L3 (a paralog of ATXN7) and anchors DUB in the core of SAGA. It was shown that SAGA is a general coactivator of RNAPII-driven transcription, which acetylates H3K9 on gene promoters and deubiquitinates H2B in the transcribed region of expressed genes (Bonnet et al., 2014). Besides a general coactivator role, SAGA is likely to have more specific functions in gene regulation (Li et al., 2017). Interestingly, inactivation of the zebrafish *atxn7* gene causes incomplete differentiation of Purkinje cells and photoreceptors, two major neuronal cell types affected in the disease (Yanicostas et al., 2012; Carrillo-Rosas et al., 2018). Recent studies in yeast indicate that *sgf73*, the ortholog of *ATXN7*, is involved in yeast longevity (calculated as the number of division a cell can achieve before going through senescence) by controlling the expression of ribosomal protein coding genes and by interacting with sirtuin2 (McCormick et al., 2014; Mason et al., 2017).

Different studies were conducted *in vitro* and in cellular and various mouse models to determine the impact of the polyQ expansion of ATXN7 on SAGA enzymatic activities and gene expression. Notably, transgenic and knockin mouse models recapitulate the retina and cerebellar pathologies observed in SCA7 patients (Yvert et al., 2000, 2001; La Spada et al., 2001; Yoo et al., 2003; Chou et al., 2010; and reviewed in Karam and Trottier, 2018) and important gene deregulations are observed in these affected tissues (La Spada et al., 2001; Abou-Sleymane et al., 2006; Helmlinger et al., 2006a; Gatchel et al., 2008; Chou et al., 2010). Several initial reports showed that mutant ATXN7 (mATXN7) is incorporated into SAGA (Palhan et al., 2005; Helmlinger et al., 2006a, b). An *in vitro* reconstituted DUB module containing mATXN7 shows normal deubiquitination activity on core histone (Lan et al., 2015), while other *in vitro* experiments lead to less consistent conclusion about the deleterious effects of mATXN7 on the KAT2A/GCN5-mediated HAT activity of SAGA (McMahon et al., 2005; Palhan et al., 2005; Helmlinger et al., 2006a; Burke et al., 2013). In SCA7 mouse models, one of the major consequence of polyQ expansion is the progressive accumulation and aggregation of mATXN7 in the nuclei of neurons, including photoreceptors and Purkinje cells (Yvert et al., 2000, 2001; La Spada et al., 2001; Yoo et al., 2003). mATXN7 is also a substrate of caspase7 and a caspase7-released fragment containing the polyQ expansion is found in patients’ cells and in the cerebellum of SCA7 mouse model (Garden et al., 2002; Young et al., 2007). Preventing proteolysis of mATXN7 by mutation in the caspase7 cleavage site reduces the aggregation and the phenotypes of SCA7 transgenic mice (Guyenet et al., 2015).

High levels of mATXN7 in the nucleus might alter SAGA function in several manners. In particular, KAT2A/GCN5, ATXN7L3, and USP22 are sequestered in mATXN7 aggregates in cultured cells (Latouche et al., 2007; Lan et al., 2015; Yang et al., 2015). Analysis of protein extracts from SCA7 knockin mouse cerebellum and astrocyte cells overexpressing mATXN7 shows that the bulk level of H2Bub is increased, suggesting that mATXN7 might cause a general dysfunction of DUB-SAGA (Lan et al., 2015; Niewiadomska-Cimicka et al., 2020). However, transcriptome comparison of astrocyte cells expressing wild type or mutant ATXN7 identifies very few differentially expressed genes (McCullough et al., 2012). Among which, the reduced expression of *RELN* consistently correlates with the reduced occupancy by mATXN7 and the increased monoubiquitinated H2B on *RELN* promoter (McCullough et al., 2012). RELN is involved in the development and maintenance of Purkinje cells (Miyata et al., 2010) and the effect of its deregulation in SCA7 pathology remains to be determined. The small subset of genes deregulated in mATXN7-expressing astrocytes is at odd with the apparent general dysfunction of SAGA-DUB caused by mATXN7.

The involvement of KAT2A/GCN5 in the SCA7 pathology was directly assessed in SCA7^100Q/100Q^ knockin mice. The deletion of one *Kat2a/Gcn5* allele accelerates the cerebellar and retinal pathology, but does not worsen gene deregulation (Chen et al., 2012), suggesting that a non-transcriptional function of KAT2A/GCN5 might contribute to SCA7 pathogenesis. Consistently, conditional deletion of *Kat2a/Gcn5* in Purkinje cells of wild type mice does not lead to severe ataxia (Chen et al., 2012). It is possible that other redundant HATs, like KAT2B/PCAF which is also present in SAGA (Nagy et al., 2009; Spedale et al., 2012), can compensate for the lack of KAT2A/GCN5. Therefore, the extent to which SAGA-HAT dysfunction contributes to Purkinje cells degeneration in SCA7 remains to be explored. As in SCA1, Purkinje cells degeneration may involve the dysfunction of RORa. Indeed, RORa transactivation activity on gene reporter system is repressed by mATXN7 in cultured cells (Strom et al., 2005), warranting further investigation of RORa dysfunction in SCA7 mouse cerebellum.

As in SCA3 mice, the SCA7 transgenic ataxin-7-Q52 mice present a deregulation of genes involved in the oligodendrocyte myelinogenesis pathway including the transcription factor *Olig1*, suggesting that SCA7 mutation also affects glial cells (Chou et al., 2010). Consistently, transgenic PrP-SCA7-92Q and Gfa2-SCA7 mouse models show clear degeneration of the Bergmann glia in the cerebellum (Custer et al., 2006). Interestingly, Purkinje cells also degenerate in these models, despite the absence of mATXN7 expression in these neurons, indicating a non-cell autonomous mechanism induced by Bergmann glia dysfunction (Custer et al., 2006). In these mice, Bergmann glia express a low level of the glutamate transporter GLAST, reducing glutamate uptake at the Purkinje cells synaptic region and leading to glutamate excitoxicity (Arundine and Tymianski, 2003; Custer et al., 2006). Study of the SCA7 transgenic mouse fxSCA7 92Q, which widely overexpresses mATXN7 in the brain, further highlights the deregulation of Purkinje cell-specific genes involved in calcium and phosphatidylinositol signaling (Stoyas et al., 2019). Promoter analysis of downregulated genes identifies an enrichment for DNA motifs bound by PGC-1α and HIF-1α transcription factors, which functions are positively regulated by the deacetylatase SIRT1 (Rodgers et al., 2005; Joo et al., 2015). Interestingly, SCA7 mice show an increased acetylation of PGC-1α and a decreased level of NAD^+^, a cofactor of SIRT1, suggesting an alteration of SIRT1 activity (Stoyas et al., 2019). Indeed, overexpression of SIRT1 in SCA7 mice restores the expression of calcium related genes, reduces the Purkinje cells pathology and enhance mice survival (Stoyas et al., 2019). Similarly, replenishment of NAD^+^ through supplemented diet improves the phenotype of SCA7 mice and restores the calcium homeostasis in neuronal progenitor cells derived from SCA7 iPSCs.

The mechanisms underlying SCA7 retinopathy have been the subject of many studies, because photoreceptors are the first to degenerate and as they represent 70% of neuronal cells in the retina, their function is easily assessed by electroretinograph activity. Electroretinograph dysfunction in SCA7 transgenic and knockin mouse models closely correlates with the progressive reduction of photoreceptor-specific genes expression essential for phototransduction and outer segment formation (Yvert et al., 2000; La Spada et al., 2001; Yoo et al., 2003; Abou-Sleymane et al., 2006; Helmlinger et al., 2006a). Consistently, SCA7 mouse photoreceptors progressively lose their outer segments (Yefimova et al., 2010), a structure entirely filled with discs of folded double membranes where phototransduction takes place. Since a proportion of outer segments is normally lost every day due to degradation, sustained expression of photoreceptor genes is essential to ensure the renewal and integrity of outer segments. The renewal process necessitates a high biosynthetic regime and a specific gene expression programs (Wright et al., 2010). Notably, the transcription factors CRX, NRL, and NR2E3 play major roles in the development, differentiation and maintenance of photoreceptors (Swaroop et al., 2010). Many CRX- and NRL-regulated genes are downregulated in SCA7 retina (La Spada et al., 2001; Abou-Sleymane et al., 2006). It was shown that CRX requires interaction with ATXN7 and SAGA to be fully functional on photoreceptor gene promoters (Palhan et al., 2005) and mATXN7 suppresses CRX transactivation activity in SCA7 transgenic mouse retina (La Spada et al., 2001; Chen S. et al., 2004). Consistently, promoters of photoreceptor-specific genes downregulated in SCA7 retina show hypoacetylation of histone H3 (Palhan et al., 2005), due to a dysfunction of the HAT activity of SAGA. Accordingly, a decreased level of RNAPII was also reported on the promoter of these genes (Helmlinger et al., 2006a). In addition to a reduced CRX transactivation activity, the expression level of CRX as well as NRL is reduced in SCA7 retina, enhancing the downregulation of their target genes (Abou-Sleymane et al., 2006; Helmlinger et al., 2006a). Another study shows that the proteotoxicity in SCA7 retina leads to hyperphosphorylation and activation of c-JUN, a transcription factor involved in the cellular stress response (Merienne et al., 2007). Interestingly, in SCA7 mice expressing a phosphorylation-resistant c-JUN, the expression level of *Nrl* is partially restored and correlates with a delay of SCA7 retinopathy. Additional analysis shows that c-JUN binds *NRL* promoter and inhibits its expression on reporter gene assay in HEK293 cells (Merienne et al., 2007).

The mouse photoreceptor has an atypical chromatin organization with a single cluster of heterochromatin surrounded by euchromatin along the nuclear membrane (Kizilyaprak et al., 2010). Photoreceptor nuclei of SCA7 transgenic and knockin mouse models have an aberrant chromatin profile with enlarged nuclei and significant chromatin decompaction (Helmlinger et al., 2006a). Chromatin decondensation might result from a decreased expression of histone H1C, which is involved in the proper compaction of photoreceptor chromatin during rod development (Popova et al., 2013). It was suggested that chromatin decompaction and nuclear enlargement lead to dilution of transcription factors and primarily affect the expression of highly expressed photoreceptor specific genes (Helmlinger et al., 2006c). Therefore, different mechanisms related to SAGA dysfunction and proteotoxicity account for the loss of gene expression and maintenance of photoreceptors in SCA7 retinopathy. It is noteworthy that TFIIF and TFIID components (but not TBP) and CBP are sequestered in mATXN7 aggregates (Yvert et al., 2001) and that ATXN7 interacts with HDAC3 in SCA7 mice (Duncan et al., 2013). However, the role of these factors and co-regulators of transcription in SCA7 gene deregulation has not been addressed so far.

A study showed that mouse ATXN7 autoregulates its expression level through a mechanism that involves the microRNA miR-124 (Tan et al., 2014). In the presence of mATXN7, SAGA dysfunction leads to lower expression of miR-124, which accelerates toxicity by over-production of mATXN7. In addition, miR-124 regulates the level of *lnc-SCA7* RNA, which itself positively influences the level of *Atxn7* mRNA by an unknown mechanism. As a consequence, the high level of *lnc-SCA7* RNA also contributes to sustain the high level of *mAtxn7* mRNA. It is proposed that these RNA crosstalks could explain the high level of mATXN7 expression in the retina and the cerebellum, accounting for the vulnerability of these tissues to the disease.

### SCA17

SCA17 presents a broad spectrum of phenotypes including ataxia, dementia, chorea, and psychiatric symptoms (Toyoshima and Takahashi, 2018). SCA17 neuropathology is characterized by atrophy of the cerebellum with major loss of Purkinje cells. Additional neurodegeneration is observed in cortical structures, caudate and putamen as well as hippocampus, parahippocampal gyrus, substantia nigra, brainstem reticular formation, and inferior olivary nucleus (Toyoshima and Takahashi, 2018).

SCA17 is caused by the expansion of more than 40 CAGs in *TBP* gene, which encodes the TATA-box-binding protein, a component of the general transcription factor TFIID. TBP has a critical function in gene transcription and knockout mice die at early embryonic stage (Martianov et al., 2002). TBP binds to basic promoter DNA motifs through its C-terminal domain, while the N-terminal extension that contains the polyQ domain modulates DNA binding and protein interactions (Basehoar et al., 2004). TBP is involved in the transcription driven by polymerases RNAPI, RNAPII, and RNAPIII (Hasegawa and Struhl, 2019) and more than 11 000 TBP binding sites in promoters of transcribed genes have been found in HEK293 cells (Hasegawa and Struhl, 2019). In particular, TBP and 13 TBP-associated proteins (TAFs) form the TFIID complex, which binds to different DNA motifs located within the gene promoter region including the TATA box. This event is followed by subsequent binding of the other general transcription factors TFIIA, TFIIB, and TFIIE (Buratowski et al., 1989) to form the transcriptional pre-initiation complex (PIC) to recruit RNAPII for transcription. For RNAPI and RNAPIII-driven transcription, TBP is associated with the multiprotein complexes SLI and TFIIIB, respectively (Learned et al., 1985; Comai et al., 1992; Huet and Sentenac, 1992).

As for the other polyQ SCAs, mutant TBP (mTBP) forms nuclear aggregates in different brain regions of SCA17 patients and SCA17 mouse models (Toyoshima and Takahashi, 2018). mTBP proteolytic cleavage generates an N-terminal fragment containing the polyQ expansion, which is involved in the formation of polyQ aggregates in SCA17 mice (Friedman et al., 2008). PolyQ expansion in mTBP impacts on gene expression through different mechanisms including aberrant protein–protein interactions, protein sequestration within aggregates and decreased binding to target DNA sequences (Friedman et al., 2007, 2008). For instance, transgenic TBP-105Q-T mice show gene deregulation, a severe motor phenotype and premature death (Friedman et al., 2007, 2008). TFIIB is sequestered in mTBP aggregates in this model. Interestingly, the expression of *Hspb1* gene (also named *Hsp27*), which encodes a chaperone involved in axonal and neuritic integrity and neuroprotective effects (Sharp et al., 2006), is significantly decreased. Downregulation of *Hspb1* correlates with the reduced occupancy of mTBP and TFIIB on its promoter. Overexpression of TFIIB or HSPB1 has protective effect and prevents neurite disruption in differentiated PC12 cell models of SCA17 (Friedman et al., 2007).

Increased production of reactive oxygen species and induction of ER stress are observed in SCA17 lymphoblastoid cell lines (Lee et al., 2014). The unfolded protein response (UPR) is a protective pathway and relies in part on the activation of XBP1, which forms complexes with different transcription factors including TBP, ATF6, and the NFY complex (Ron and Walter, 2007; Hetz, 2012). There are evidences that the UPR is compromised in SCA17. For example, NFYA, a regulatory unit of NFY complex, is sequestered in mTBP aggregates in SCA17 transgenic mice and cultured cells, and is thus decreased on target promoters, such as *HSPA5*, leading to reduced ER stress response (Huang et al., 2011; Lee et al., 2012). Furthermore, the XBP1-regulated *Manf* gene, coding for a neuroprotective factor (Hao et al., 2017), is strongly downregulated in the cerebellum of SCA17 TBP-105Q knockin mice (Yang et al., 2014). In cultured cells expressing mTBP, XBP1 shows a decreased binding to the *MANF* promoter. Interestingly, SCA17 TBP-105Q knockin mice that overexpress *MANF* in the brain show a significant improvement of motor phenotypes and a decrease of Purkinje cell degenerative signs (Yang et al., 2014). MANF protective effect on Purkinje cells appears to involve the protein kinase C signaling pathway. Therefore, mTBP appears to alter the transactivation activity of XBP1 and NFY, causing downregulation of their target genes involved in the ER stress response and signaling pathways. Interestingly, a FDA approved drug called piperine is found to induce MANF activity and to improve motor performance of SCA17 knockin mice (Guo et al., 2018). Similarly, treatment with Shaoyao Gancao Tang reduces mTBP aggregation, increases expression of *NFYA* and *HSPA5* as well as *PGC-1α* and *NRF2*, and improves motor phenotypes of SCA17 transgenic mice (Chen et al., 2019).

Alteration of neurite outgrowth in different cellular and mouse models of SCA17 correlates with decreased expression of the *Trka* gene encoding the receptor of the nerve growth factor (Chao and Hempstead, 1995; Shah et al., 2009). Analysis of *Trka* promoter sequence identifies a binding site for the Specificity Protein 1 (SP1) (Shah et al., 2009), which is known to interact with TBP (Emili et al., 1994). In cultured cells overexpressing mTBP, SP1 is not sequestered in aggregates, but shows a decreased transactivation activity in reporter gene assay (Shah et al., 2009). In the TBP-105Q knockin mice, abnormal activity of SP1 causes a decreased level of INPP5A, a protein strongly enriched in Purkinje cells and involved in the degradation of IP3 (Liu et al., 2020). Importantly, downregulation of *Inpp5a* in TBP-105Q knockin mice increases the IP3 levels and leads to Purkinje cells degeneration, while overexpression of *Inpp5a* through AAV viral vector decreases IP3 level and reduces Purkinje cells loss.

Yang et al. (2017) used mouse genetic approach to study the effect of mTBP expression in neuronal and glial cells on the phenotype. While expression of mTBP in neurons and astrocytes alone has mild effect, the expression in both cell types exacerbates mouse phenotypes and Purkinje cell degeneration. Furthermore, using co-culture of primary astrocytes and neurons, the authors showed that mTBP-expressing astrocytes activate inflammatory signaling pathways and cause neuronal degeneration, which is prevented by blocking the nuclear signal kB (NF-kB) pathway. This study nicely illustrates that the synergistic toxicity of mTBP in neuronal and glial cells is critical for SCA17 pathogenesis.

Patients with long CAG repeats present a severe phenotype with muscle weakness (Maltecca et al., 2003). SCA17 floxed TBP-105Q knockin mice where mTBP is only expressed in muscle shows muscle degeneration with fragmented muscle morphology and centrally positioned nuclei (Huang S. et al., 2015). Gene expression analysis reveals a strong deregulation of muscle specific genes such as *Calc-l*, *Myo-bpc2*, *Ckm*, or *Trim72* and decreased level of MYOD, a key transcription factor required for a proper differentiation and maintenance of myoblasts (Davis et al., 1987) and known interactor of TBP (Heller and Bengal, 1998). Analysis of the muscle cell line C2C12 expressing mTBP indicates that MYOD has decreased occupancy on promoters of target muscle genes such as *Mhc4* and *Mck*. Importantly, overexpression of MYOD alleviates muscle degeneration in TBP-105Q knockin mice, further confirming its central role in SCA17 muscle pathology (Huang S. et al., 2015).

## Perspectives and Opportunities

### Converging Disease Pathways and Shared Pathomechanisms

Despite shared genetic, clinical, and neuropathological features of polyQ SCAs, we still need a deeper understanding of the points of intersection and divergence of the pathophysiology of these diseases. A number of reviews have already compared the major features of cerebellar degeneration found in animal models with ataxic symptoms and pointed out commonly altered signaling pathways, especially affecting the physiology of Purkinje cells (Meera et al., 2016; Hoxha et al., 2018). The system biology approaches have examined human cerebellar gene expression data and identified modules of cerebellar co-expressed genes correlating with ataxia (Bettencourt et al., 2014). Recently, new knockin mouse models of polyQ SCAs, which recapitulate the expression of the disease mutation in accurate spatio-temporal manner in tissues, appeared to be excellent tools for more accurate exploration of the pathophysiology of polyQ SCAs. In the future, the comparison of differentially expressed genes in each polyQ SCA, for a particular tissue or cell type at equivalent disease stage, should provide opportunities to uncover the shared pathogenic signatures, paving a way to novel therapeutic strategies that could prove beneficial for multiple SCAs.

**FIGURE 1:**
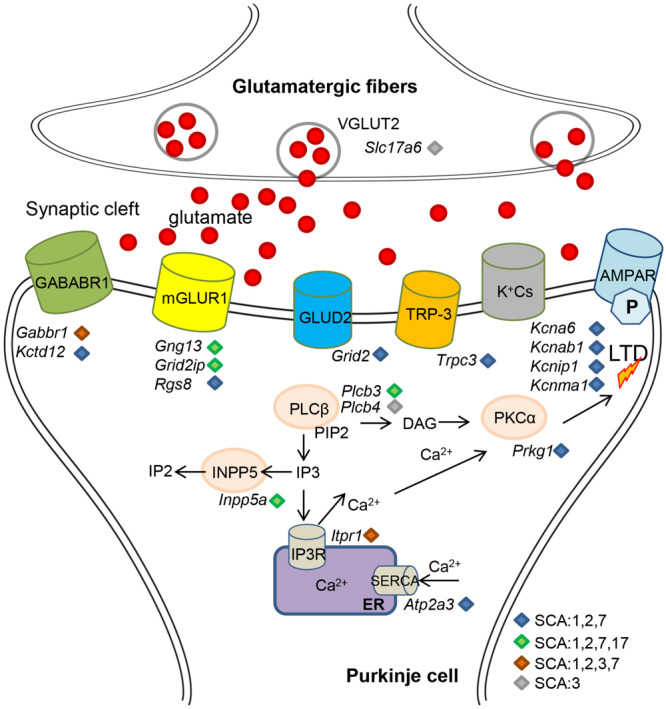
Schematic of Purkinje cell enriched gene deregulations in polyQ SCAs affecting glutamatergic synapse. Deregulations of gene expression in Purkinje cells result in alterations of the protein level of ion channels, glutamate transporters, and receptors together with downstream effectors. Production of secondary messengers and calcium homeostasis are impaired, which leads to alteration of long-term depression and pacemaker activities.

Several converging disease pathways in polyQ SCAs have already been identified. Comparison of gene expression data revealed common deregulation of the IGF signaling pathway in SCA1 and SCA7 knockin mouse models (Gatchel et al., 2008). In addition, WGCNA analysis of differentially expressed genes in SCA1 and SCA2 mice identified similar modules and hubs of Purkinje cell-enriched genes and highlighted common altered pathways such as LTD, phosphatidylinositol, calcium, and cell signaling (Lin et al., 2000; Serra et al., 2004; Ingram et al., 2016; Pflieger et al., 2017). Many of the reported Purkinje cell-enriched genes were also deregulated in SCA3, SCA7, and SCA17 (Figure 1; Chou et al., 2008, 2011, 2014; Stoyas et al., 2019; Liu et al., 2020). Direct comparison of polyQ SCA gene deregulation is thus warranted to highlight essential points of intersection in the pathogenesis of polyQ SCAs. For instance, the relevance of *Inpp5a* downegulation in Purkinje cells degeneration has already been tested in SCA17 (Liu et al., 2020) and is likely to be demonstrated in other polyQ SCAs as well. Other converging disease pathways are implicated in the activation of immune response genes including interferon response and chronic neuroinflammation, and are prominent in SCA1, SCA3, SCA6 SCA7, and SCA17 as well as HD (Merienne et al., 2007; Chort et al., 2013; Aikawa et al., 2015; Yang et al., 2017; Driessen et al., 2018; Palpagama et al., 2019; Liu et al., 2020; Niewiadomska-Cimicka et al., 2020). Cross analysis of polyQ SCAs will help to understand how and when these injury responses are activated and their exact roles in the pathogenesis. One emerging theme that appears common in mouse models of SCA2, SCA3, SCA7, and other polyQ disorders is the deregulation of genes involved in myelination of oligodendrocytes and maintenance of their functions (Chou et al., 2010; Huang B. et al., 2015; Ramani et al., 2017; Sen et al., 2019a, b). Since patients show clear atrophy of the white matter in different brain regions, the mechanisms underlying the pathology of glial cells deserve further investigation. There is thus a need for a higher, namely cell-type specific resolution of gene expression studies of polyQ SCA models. Single-cell transcriptome approaches are becoming more standardized (Lake et al., 2016; Saunders et al., 2018) and open new opportunities. Other approaches allow the analysis of cell-type specific gene deregulations without the need for transgenic animals (Xu et al., 2018), which can be extremely powerful. Challenging the spatial organization of transcriptomes is currently the next step for the precise understanding of how cerebellar circuits are affected. Recent technological break-through allows to follow gene expression changes in a high spatial resolution in tissues (Eng et al., 2019; Rodriques et al., 2019).

**FIGURE 2:**
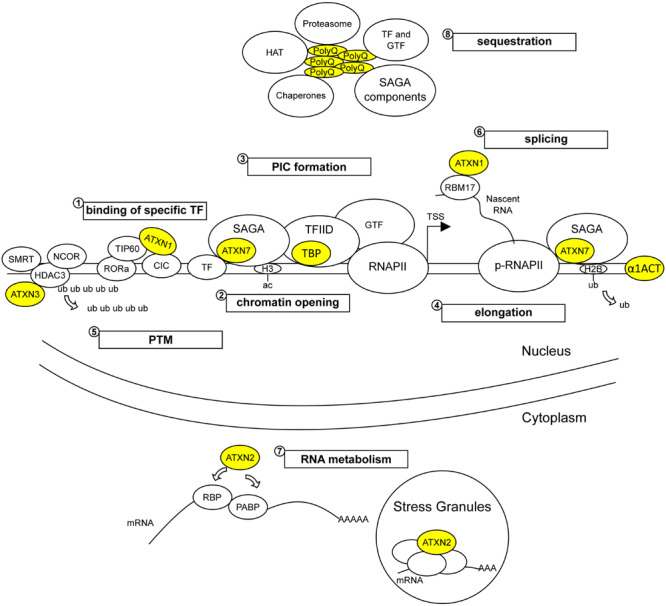
Wild type and pathological functions of polyQ SCA proteins in gene expression. Wild type SCA proteins participate to various regulatory steps of gene expression, such as binding to DNA sequences (α1ACT and TBP), modulating repression or activation activities of transcription factors (TF) (ATXN1, ATXN3, ATXN7, and TBP), modifying epigenetic marks on promoters (ATXN7/SAGA and ATXN3/HDAC3/SMRT/NCOR complexes), assembling pre-initiation complex (PIC) (TBP/TFIID) that includes general transcription factors (GTFs), deubiquitinating substrates (ATXN3 and ATXN7/SAGA) and participating to RNA metabolism (ATXN1 and ATXN2). Due to abnormal interactions with their native partners, mutant SCA proteins alter (1) the activation or repressor activities of TFs, (2) the epigenetic marks of histones involved in chromatin opening, (3) the formation of PIC recruiting RNAPII on gene promoters, and (4) the transcription elongation mediated by phosphorylated RNAPII (p-RNAPII) and by histone modification on the gene body. Mutant SCA proteins also alter (5) the post-translation modification (PTM) of nuclear proteins, (6) RNA splicing and (7) other RNA processing such as translation. In addition, (8) mutant SCA proteins form soluble oligomers and insoluble aggregates that sequester native protein partners and proteins involved in nuclear proteostasis, accounting for perturbation of gene expression. ub, ubiquitin; ac, acetyl; RBP, ribosome binding proteins.

Further comparative studies on factors involved in transcription might help to shed light into shared pathomechanisms of gene deregulation induced by polyQ expansion in disease proteins (Figure 2). For example, SCA1, SCA2, and SCA7 gene expression data already identified the deregulation of common RORa-target genes in mouse models (Serra et al., 2006; Pflieger et al., 2017). RORa is an important transcriptional activator of genes involved in calcium homeostasis and the maintenance of Purkinje cells (Hamilton et al., 1996; Serra et al., 2006). It was shown that mATXN1 destabilizes RORa transactivation activity through aberrant interaction with TIP60 (Serra et al., 2006). A possible interconnection between the mechanisms whereby mATXN1, mATXN2, and mATXN7 cause RORa dysfunction represents an interesting hypothesis. Along this same line, cumulative evidences indicate that like TFIID, the SAGA complex can deliver TBP to gene promoters (Bhaumik and Green, 2002). SAGA and TFIID share five TAFs and both complexes are required for global gene expression in yeast (Baptista et al., 2017; Warfield et al., 2017). Recent structural examination of SAGA associated with TBP provides additional information about the deposition of TBP on gene promoters (Papai et al., 2020). Therefore, comparison of cerebellar or cell-type specific transcriptomes of SCA7 and SCA17 knockin mouse models is likely to unveil common mechanisms of gene deregulation and provide further insight into the natural function of SAGA and TFIID in mammalian cells. An additional line of converging mechanism concerns the alteration of nuclear proteotasis. A consistent observation in mouse models of polyQ disorders is an early and progressive accumulation of the disease protein in the nucleus. This suggests that mechanisms of PQC in the nucleus become rapidly overwhelmed by the increased abundance of misfolded proteins. Compared to other cellular compartments, the nucleus has a relatively distinct ubiquitin-proteasome system (Jones and Gardner, 2016) and lacks macroautophagy, a mechanism involved in aggregate clearance in the cytoplasm. Several players of PQC such as chaperones and proteasomes are sequestered in polyQ aggregates (Figure 2) and might be depleted for their interactions with other nuclear proteins. As proposed earlier (Shibata and Morimoto, 2014), perturbation of nuclear proteostasis network by chronic proteotoxic stress may impact on diverse nuclear functions including gene expression and epigenetics and contribute to protein conformation diseases. Since causative SCA proteins associate with various interactors (Table 1), accumulation of mutant monomeric, oligomeric, or truncated polyQ proteins in the nucleus is likely to alter (sub)complex stoichiometry or cause aberrant or promote novel deleterious interactions.

### Broader Aspects of Gene Deregulation on the Cellular Pathology

Precise regulation of gene expression is essential for neuronal development, function, and maintenance. Transcription of protein coding genes requires the coordinated action between specific DNA elements, transcription factors and epigenetic modulators. Subsequent steps of transcriptional elongation, RNA processing, export, translation and degradation, further shape what cells get as net products. Another orchestrating mechanism controlling the gene expression is a crosstalk between main transcriptional apparatus and non-coding RNAs (ncRNA) (e.g., microRNAs, long ncRNA, enhancer RNA, etc.). Wild type SCA proteins are involved in multi-level gene expression regulation (Figure 2), which are the areas to identify the pathomechanisms underlying gene deregulation in polyQ SCAs. However, except for microRNAs (see review Dong and Cong, 2019), many aspects of gene deregulation remain poorly explored in the context of polyQ SCAs and warrant further investigation.

Few studies have investigated the epigenetic aspects of gene deregulation of polyQ SCAs (Table 2). Histone modifications such as acetylation or ubiquitination, have often been analyzed at the bulk level or on selected promoters, while genome-wide analysis using ChIP followed by high-throughput sequencing (ChIP-seq) is likely to provide a more complete view on the role of epigenetic marks in the disease. For instance, the studies of HD mouse models have shown that histone deacetylation and transcriptional deregulation correlated for only a subset of deregulated genes, which, however, have high significance for the pathology (Valor et al., 2013). In another case, hypoacetylated promoters of neuronal identity genes correlated with increased susceptibility to transcriptional deregulation and to association with additional epigenetic impairments of trimethylation of lysine 4 of histone H3, a mark of active transcription (Guiretti et al., 2016).

Several studies have established that superenhancers, which are large clusters of enhancers characterized by hyperacetylation of lysine 27 of histone H3 (H3K27ac) are involved in the expression of cell identity genes (Hnisz et al., 2013; Whyte et al., 2013). A recent study identified many neuronal genes expressed in the mouse striatum associated with H3K27 hyperacetylated regions characteristic of superenhancers (Achour et al., 2015). In HD mice, striatal genes, which were associated with these putative superenhancer regions, bore H3K27 hypoacetylation and were preferentially downregulated, suggesting that affected neurons in HD progressively lose the expression of neuronal identity genes (Achour et al., 2015). One HAT responsible for H3K27ac is CBP, which is sequestered in polyQ aggregates in HD as well as in polyQ SCAs (McCampbell et al., 2000; Nucifora et al., 2001; Yvert et al., 2001; Jiang et al., 2003). This could suggest that neurons vulnerable in polyQ SCAs might also preferentially lose the expression of neuronal identity genes. Purkinje cells in most polyQ SCAs indeed showed decreased expression of cell-type specific genes (Figure 1). Along this line, we have previously proposed that photoreceptors in SCA7 mice progressively lose their neuronal identity based on several observations (Niewiadomska-Cimicka and Trottier, 2019). Notably, results from several studies indicated that SCA7 photoreceptor dysfunction correlates with the early and progressive reduced expression of most photoreceptor-specific genes important for phototransduction and outer segment formation (Yvert et al., 2000; La Spada et al., 2001; Yoo et al., 2003; Chen S. et al., 2004; Palhan et al., 2005; Abou-Sleymane et al., 2006; Helmlinger et al., 2006a; Yefimova et al., 2010). Moreover, inhibition of zebrafish *atxn7* leads to incomplete differentiation of photoreceptors (Yanicostas et al., 2012; Carrillo-Rosas et al., 2018). These observations in SCA vulnerable cells thus call for additional genome-wide analyses of epigenetic marks associated with the expression of neuronal cell identity genes.

Finally, impaired epigenetic programs may link the pathogenesis of polyQ SCAs and aging. Aging is recognized as a significant risk factor for polyQ SCAs and other neurodegenerative disorders and the link between aging, epigenetic and chromatin alterations is now established (reviewed in Sen et al., 2016). Aged mice show alterations of histone acetylation/methylation levels, leading to an increase in repressive marks and a decrease in activating marks. The question whether age-dependent epigenetic alterations superimpose to other disease-related modifications and contribute to neuronal demise is now being addressed in HD and other neurodegenerative disorders (Berson et al., 2018).

Global gene expression studies in polyQ SCA mouse models have generally pointed out the deregulation of a relatively small number of genes in a given tissue or cell type. This is consistent with our current understanding of pathomechanisms whereby polyQ SCA proteins affect a discreet set of transcription factors or co-regulators leading to deregulation of a specific subset of genes, which appear crucial for the function and survival of targeted neurons. However, one should keep in mind that classic molecular techniques only measure the steady-state level of RNA molecules. The recently uncovered phenomenon of transcription buffering (reviewed in Helenius et al., 2011; Timmers and Tora, 2018), allows a certain level of cross-talk between cytoplasm and nucleus to adjust and maintain constant mRNAs level in yeast. In particular, yeast studies showed that inactivation of subunits of TFIID (Warfield et al., 2017) or SAGA (Baptista et al., 2017) lead to a global decrease of transcription, but was followed by transcription buffering. Transcription buffering may thus mask more global alterations of gene transcription in SCA7, SCA17, and possibly in other polyQ SCAs. Therefore, re-evaluation of transcription dysregulation by the analysis of nascent RNA (Mayer et al., 2015) or of genome-wide patterns of phosphorylated RNAPII (Helenius et al., 2011) may provide new insight into the pathomechanisms underlying polyQ SCAs.

### Restoring Proper Gene Expression

A number of strategies have been developed to restore proper gene expression in preclinical models of polyQ diseases and led to improve phenotypes (Xiang et al., 2018). As previously discussed, alterations of histone acetylation in SCA3 models led to several attempts to correct gene deregulation and disease phenotype by using different HDACi. However, it remains unclear whether the beneficial effects of treatments are related to the correction of gene expression or to other neuroprotective effects of the drugs (Chou et al., 2011; Esteves et al., 2015; Wang et al., 2018). Similarly, treatment of astrocytes expressing mATXN7 with the HDACi led to partial restoration of *RELN* gene expression by promoting the aggregation of mATXN7 into large aggregates and preventing its deleterious effect on *RELN* promoter (McCullough et al., 2012). It is also noteworthy that HDAC3 haploinsufficiency does not rescue the phenotype of SCA1 mice (Venkatraman et al., 2014), possibly due to the important role of HDAC3 in Purkinje cells function and survival (Venkatraman et al., 2014). Therefore, the use of HDACi in treatment of polyQ SCAs is currently hampered by our limited knowledge of HDAC roles on neuronal physiology and the pleiotropic effects of the drugs (Didonna and Opal, 2015). Since downregulation of Purkinje cells genes in SCA7 mice was associated to the reduced function of SIRT1, an approach was developed to restore SIRT1 function using NAD+ repletion to improve the phenotype (Stoyas et al., 2019). SIRT1 is also a target for HD and other neurodegenerative disorders and different strategies are being developed to potentiate its function (Wang et al., 2016; Williams et al., 2017). Interestingly, the development of RNA silencing strategies to reduce mutant protein expression in mouse models has led to concomitant improvement to gene expression and disease phenotypes in SCA1, SCA2, and SCA7 (Scoles et al., 2017; Friedrich et al., 2018; Niu et al., 2018), further highlighting the proxy of gene deregulation with the etiology of polyQ SCAs.

## Author Contributions

AN-C, AH, and YT wrote the manuscript and approved the final manuscript.

## Conflict of Interest

The authors declare that the research was conducted in the absence of any commercial or financial relationships that could be construed as a potential conflict of interest.
